# Time-dependent responses of brain injury biomarkers after severe cerebral desaturation in paediatric patients during cardiac surgery with cardiopulmonary bypass: a single-centre observational cohort study

**DOI:** 10.3389/fcvm.2026.1879937

**Published:** 2026-07-29

**Authors:** Zeng-Chun Wang, Xiang Lin, Ling-Shan Yu, Qi-Liang Zhang, Ye Chen, Wenfeng Chen

**Affiliations:** 1 Department of Cardiac Surgery, Fujian Maternity and Child Health Hospital, Fuzhou, China; 2 Department of Cardiac Surgery, Fujian Children’s Hospital (Fujian Branch of Shanghai Children’s Medical Center), Fuzhou, China; 3 College of Clinical Medicine for Obstetrics & Gynecology and Pediatrics, Fujian Medical University, Fuzhou, China; 4 Institute of Life Sciences, College of Biological Science and Engineering, Fuzhou University, Fuzhou, China

**Keywords:** cardiopulmonary bypass, cerebral desaturation, congenital heart disease, near-infrared spectroscopy, neuron-specific enolase, regional cerebral oxygen saturation

## Abstract

**Background:**

Neurological injury remains an important concern in paediatric patients after congenital heart disease (CHD) surgery with cardiopulmonary bypass (CPB). Regional cerebral oxygen saturation (rScO_2_) can be used to identify unstable intraoperative cerebral oxygenation, but its association with the postoperative responses of brain injury biomarkers remains unclear.

**Methods:**

This single-centre observational cohort study included 104 children who underwent congenital heart surgery with CPB. The maximum intraoperative decrease in rScO_2_ was calculated as the preoperative value minus the lowest value recorded during bypass, during aortic cross-clamping, or after the return of spontaneous circulation. Severe cerebral desaturation was defined as a decrease of at least 20%. The levels of inflammatory cytokines and biomarkers of brain injury were measured preoperatively and on postoperative Days 0 (POD0), POD1, and POD2. Postoperative changes were calculated as log_2_ fold changes from baseline and z-standardized. The composite inflammation score derived from tumour necrosis factor-alpha (TNF-α), interleukin-1 beta (IL-1β), interleukin-6 (IL-6), and interleukin-8 (IL-8) levels, and the composite brain injury score derived from glial fibrillary acidic protein (GFAP), S100 calcium-binding protein B (S100B), and neuron-specific enolase (NSE) levels were averaged to derive the composite brain injury score. Adjusted associations were evaluated using multivariable linear regression models, linear mixed-effects models (LMMs), and generalized estimating equations (GEEs), with the non-severe group serving as the reference category.

**Results:**

Children in the severe group were younger than those in the non-severe group, but most of the perioperative characteristics were similar. Severe desaturation was not associated with the composite inflammation score. In contrast, severe desaturation was independently associated with a higher postoperative composite brain injury score in both the LMM and the GEEs. The strongest difference between the severe and non-severe groups occurred on POD1 and remained significant after false discovery rate correction. Single-marker analyses showed directionally positive postoperative responses for GFAP, S100B, and NSE.

**Conclusions:**

A maximum intraoperative decrease in rScO_2_ of at least 20% was associated with postoperative responses of brain injury biomarkers in children who underwent congenital heart surgery with CPB. The association was most evident on POD1 and attenuated by POD2.

## 1 Introduction

Congenital heart disease (CHD) is one of the most common birth defects in children, with an incidence of approximately 1% (1, 2). With continuous advances in surgical techniques, anaesthetic management, and perioperative monitoring, perioperative mortality in children with CHD has markedly decreased (3). As survival has improved, the clinical focus has increasingly shifted towards preserving brain health and long-term neurodevelopment (4). The brain tissue of infants and young children is still immature and more susceptible to ischaemia, hypoxia, and inflammatory stimuli (5). Therefore, the early identification and risk assessment of perioperative brain injury have received increasing attention. Regional cerebral oxygen saturation (rScO_2_) can be monitored using near-infrared spectroscopy. This method provides a continuous and non-invasive bedside assessment. rScO_2_ reflects the balance between the cerebral oxygen supply and demand and is clinically useful for identifying abnormal oxygen delivery during the perioperative period (6, 7).

Cardiopulmonary bypass (CPB) can trigger perioperative inflammatory responses and brain injury (8, 9). Studies have shown that the incidence of systemic inflammatory response syndrome in paediatric patients within 72 h after congenital heart surgery is as high as 34.5% (8, 10). Moreover, because children with CHD often have poor baseline cerebral oxygenation and because the cerebral metabolic rates of neonates and infants are approximately 30% higher than those of adults, children are more sensitive to inflammatory stimulation and are more likely to develop severe brain injury under the combined effects of microembolism, tissue hypoperfusion, and other factors (5, 11). Cytokines such as tumour necrosis factor-alpha (TNF-α), interleukin-1 beta (IL-1β), interleukin-6 (IL-6), and interleukin-8 (IL-8) can reflect the degree of systemic inflammatory activation.

In parallel, biomarkers of brain injury, such as glial fibrillary acidic protein (GFAP), S100 calcium-binding protein B (S100B), and neuron-specific enolase (NSE), can provide complementary information about postoperative cerebral injury biology (12–14). GFAP reflects astroglial injury, S100B is associated with glial activation and blood–brain barrier disruption, and NSE is commonly used as a neuronal injury biomarker (13–17). Previous studies have explored cerebral oximetry and serum neuromarkers in paediatric patients undergoing cardiac surgery, but the relationship between a large intraoperative decrease in rScO_2_ and integrated postoperative responses of brain injury biomarkers has not been fully characterized (18, 19).

From a pathophysiological standpoint, intraoperative cerebral oxygen desaturation is thought to contribute to postoperative brain injury via multiple pathways, including ischaemic–hypoxic stress, reperfusion injury, and the activation of inflammatory cascades (20, 21). However, in real-world clinical practice, the evidence remains insufficient to confirm that this causal chain is consistently operative or that intraoperative decreases in rScO_2_ correlate clearly and reproducibly with postoperative inflammatory responses and changes in brain injury biomarkers. On the one hand, ethics and clinical practice limit randomized controlled studies based on the active manipulation of cerebral oxygen levels (22); on the other hand, perioperative inflammation and brain injury are often jointly influenced by multiple factors, including surgical trauma, cardiopulmonary bypass management, the complexity of the underlying disease, and individual physiological reserve. Therefore, whether intraoperative cerebral oxygen desaturation more closely reflects the systemic inflammatory burden or is better regarded as a neurological risk signal remains to be further clarified.

In this single-centre observational cohort study, we evaluated whether the maximum intraoperative decrease in rScO_2_ from the pre-CPB value was associated with the postoperative responses of inflammatory cytokines and brain injury biomarkers in children who underwent congenital heart surgery with CPB. We defined severe cerebral desaturation as a maximum absolute decrease of at least 20% and analysed postoperative responses using composite inflammation and brain injury scores. Because these measurements were repeated across postoperative days, we used both linear mixed-effects models (LMMs) (23) and generalized estimating equations (GEEs) (24) to estimate adjusted differences between the severe and non-severe groups, with the non-severe group used as the reference category.

## 2 Materials and methods

### 2.1 Study design and participants

This single-centre observational cohort study screened children who underwent cardiac surgery with CPB between October 2024 and November 2025. The inclusion criteria were as follows: aged younger than 18 years, a diagnosis of CHD, and planned cardiac surgery with CPB. Patients were excluded if they had severe preoperative comorbidities, such as hepatic failure or renal failure; had undergone cardiopulmonary resuscitation before surgery; had preoperative neurological or intracranial comorbidities, such as intracranial infection or epilepsy; died after surgery; had unsuitable blood samples; had incomplete clinical data; or if their parents or legal guardians declined to participate in this study. Finally, 104 children were included in the analysis. Perioperative clinical data, including sex, age, height, weight, Risk Adjustment for Congenital Heart Surgery-1 score, duration of surgery, cardiopulmonary bypass time, aortic cross-clamp time, duration of anaesthesia, and perioperative levels of regional cerebral oxygen saturation, inflammatory cytokines, and brain injury biomarkers, were collected. This study was conducted in accordance with the Declaration of Helsinki and was approved by the Ethics Committee of Fujian Children's Hospital (protocol code 2023ETKLR05087; approved on 12 May 2023). Written informed consent was obtained from the parents or legal guardians of all participants before enrolment.

### 2.2 Cerebral oximetry and exposure definition

An INVOS™ 5100C (Medtronic) cerebral and regional oximetry monitor was used to continuously monitor rScO_2_ in children with CHD. After the induction of anaesthesia, near-infrared spectroscopy sensors were placed on the skin over both sides of the forehead, and intraoperative changes in rScO_2_ were recorded continuously. rScO_2_ was recorded at four perioperative periods: before CPB (pre-CPB) (from the start of surgery to the start of CPB), during CPB (from the start of CPB to aortic occlusion), during aortic cross-clamping (ACC) (from aortic occlusion to aortic opening), and after the return of spontaneous circulation (ROSC) (from the return of the heartbeat to the end of the operation). The primary exposure was the maximum absolute intraoperative decrease from the pre-CPB value. The variables were defined as follows:

ΔrScO_2_-CPB = rScO_2_, Pre-CPB - rScO_2_, During CPB
ΔrScO_2_-ACC = rScO_2_, Pre-CPB - rScO_2_, During ACC
ΔrScO_2_-ROSC = rScO_2_, Pre-CPB - rScO_2_, After ROSC
ΔrScO_2_-max = rScO_2_, Pre-CPB - min(rScO_2_, During CPB, rScO_2_, During ACC, rScO_2_, After ROSC)

Previous studies have rarely explored the relationship between the decrease in rScO_2_ in paediatric patients during cardiac surgery and the postoperative response of biomarkers of brain injury. This exploratory study initially explored this relationship. Due to the limited sample size, supplementary analyses that treated ΔrScO_2_-max as a continuous variable could not be considered. After referring to numerous previous studies, the majority of centres and studies consider that a ≥20% decrease from baseline is generally considered a relative threshold (25, 26). Accordingly, we used a 20% decrease as the threshold. In this study, severe intraoperative cerebral desaturation was defined as a maximum decrease in rScO_2_ of ≥20% (ΔrScO_2_-max ≥20%), whereas a decrease of <20% was classified as non-severe desaturation.

### 2.3 Measurement of the levels of inflammatory cytokines and brain injury biomarkers

The inflammatory cytokines included TNF-α, IL-1β, IL-6, and IL-8, while the brain injury biomarkers included GFAP, S100B, and NSE. Since systematic data on postoperative neurological clinical outcomes and long-term neurodevelopmental follow-up data were not yet available, perioperative levels of inflammatory cytokines and brain injury biomarkers were analysed in this study as surrogate risk signals. Blood samples were collected preoperatively (Pre), immediately after surgery (within 1 h, POD0), on postoperative Day 1 (24 h after the operation) (POD1) and postoperative Day 2 (48 h after the operation) (POD2). Serum was isolated after centrifugation, and the levels of the corresponding markers were measured using the following enzyme-linked immunosorbent assay (ELISA) kits (ZCIBIO Technology): ZC-35733 (human TNF-α), ZC-32420 (human IL-1β), ZC-32446 (human IL-6), ZC-32449 (human IL-8), ZC-34594 (human GFAP), ZC-32056 (human S100B), and ZC-34626 (human NSE). Measurements were performed using an MD-SpectraMax 190 microplate reader (Molecular Devices).

### 2.4 Composite scores of inflammatory cytokines and brain injury biomarkers

For each inflammatory cytokine and brain injury biomarker, postoperative fold changes in its level were calculated relative to the preoperative level and reported as log_2_FC values.

$$\mathrm{lo}g_{2}\mathrm{FC}=\mathrm{lo}g_{2}(\mathrm{postoperative}\;\mathrm{value}/\mathrm{preoperative}\;\mathrm{value})$$

Each biomarker-specific log_2_FC was standardized to a z score at each postoperative time point using the following equation to allow inflammatory cytokines and brain injury biomarkers with different units and distributions to be combined:

$$\;z\;\mathrm{score}=(\mathrm{individual}\;\mathrm{lo}g_{2}\mathrm{FC}-\mathrm{group}\;\mathrm{mean}\;\mathrm{lo}g_{2}\mathrm{FC})/\mathrm{group}\;\mathrm{SD}\;\mathrm{of}\;\mathrm{lo}g_{2}\mathrm{FC}$$

Composite scores were then calculated by averaging the z scores of the levels of inflammatory cytokines and brain injury biomarkers within the same biological system. The composite score for inflammation was based on TNF-α, IL-1β, IL-6, and IL-8 levels. The composite score for brain injury was based on the levels of GFAP, S100B, and NSE. Higher composite scores indicated greater overall inflammatory or brain injury biomarker activity.

Compositescore=meanzscoreswithineachgroup

For cross-sectional summary analyses, the mean and peak composite brain injury scores were also calculated across POD0 and POD2. The mean score represented the average postoperative brain injury biomarker activity. The peak score represented the highest observed postoperative activity of biomarkers of brain injury.

MeanScore=mean(Score,POD0,Score,POD1,Score,POD2)

PeakScore=max(Score,POD0,Score,POD1,Score,POD2)

### 2.5 Statistical analysis

For all adjusted analyses, severe cerebral desaturation was defined as a ΔrScO_2_-max ≥20%. The non-severe group was used as the reference category. Therefore, the coefficient for severe desaturation represents the adjusted difference in the outcome between the severe and non-severe groups.

Single-time-point and summary brain injury outcomes were analysed using multivariable linear regression models with heteroskedasticity (unequal error variances)-consistent HC3 robust standard errors. The outcomes were the composite brain injury scores at POD0, POD1, and POD2; the mean and peak composite brain injury scores across POD0–POD2; and the log_2_ fold changes in the levels of individual brain injury biomarkers. Models were adjusted for age, body weight, sex, Risk Adjustment for Congenital Heart Surgery-1 (RACHS-1) score, and CPB time. Spearman's rank correlation analysis was performed as an unadjusted descriptive analysis for the composite brain injury outcomes.

$$B_{i,k}=\alpha_{0}+\alpha_{1}D_{i}+\alpha_{2}\mathrm{Ag}e_{i}+\alpha_{3}\mathrm{Weigh}t_{i}+\alpha_{4}\mathrm{Mal}e_{i}+\alpha_{5}\mathrm{RACH}S_{i}+\alpha_{6}\mathrm{CPBtim}e_{i}+\varepsilon_{i}$$

In this model, B_i,k_ denotes the composite brain injury outcome k for patient i, including POD0, POD1, POD2, mean, and peak composite scores. D_i_ denotes severe cerebral desaturation, with D_i_ = 1 for ΔrScO_2_-max ≥20% and D_i_ = 0 for the non-severe reference group. The coefficient α_1_ is the adjusted difference between the severe and non-severe groups. Age_i_, Weight_i_, Male_i_, RACHS_i_, and CPBtime_i_ denote age, body weight, male sex, RACHS-1 score, and cardiopulmonary bypass time, respectively; α_2_ to α_6_ are their corresponding regression coefficients. The term ε_i_ is the residual error.

Exploratory analyses of single markers used the same covariate structure, HC3 robust standard errors, and reference category coding. The outcomes included the log_2_ fold changes in GFAP, S100B, and NSE levels at POD0, POD1, and POD2 and the mean and peak log_2_ fold changes across POD0–POD2.

$$M_{i,m,k}=\alpha_{0}+\alpha_{1}D_{i}+\alpha_{2}\mathrm{Ag}e_{i}+\alpha_{3}\mathrm{Weigh}t_{i}+\alpha_{4}\mathrm{Mal}e_{i}+\alpha_{5}\mathrm{RACH}S_{i}+\alpha_{6}\mathrm{CPBtim}e_{i}+\varepsilon_{i}$$

Here, M_i,m,k_ denotes the log_2_ fold change in the levels of biomarker m and outcome metric k in patient i. The biomarker m refers to GFAP, S100B, or NSE. The metric k refers to the POD0, POD1, POD2, mean, or peak log_2_ fold change. All the other parameters have the same meanings as in the composite brain injury model.

Repeated-measures analyses used postoperative composite inflammation and brain injury scores at POD0, POD1, and POD2 as outcomes. LMMs included patient identity (ID) as a random intercept to account for within-patient correlations. The overall linear mixed-effects model (LMM) estimated the average adjusted difference between the severe and non-severe groups across postoperative time points.

$$\mathrm{Scor}e_{i,s,t}=\beta_{0}+\beta_{1}D_{i}+\beta_{2}I(\mathrm{POD}1_{t})+\beta_{3}I(\mathrm{POD}2_{t})+\beta_{4}\mathrm{Ag}e_{i}+\beta_{5}\mathrm{Weigh}t_{i}+\beta_{6}\mathrm{Se}x_{i}+\beta_{7}\mathrm{RACH}S_{i}+\beta_{8}\mathrm{CPBtim}e_{i}+u_{i}+\varepsilon_{i,t}$$

In this model, Score_i,s,t_ denotes the composite score for patient i, biological system s, and postoperative time point t. The system s refers to either inflammation or brain injury, and t refers to POD0, POD1, or POD2. POD0 was used as the reference time point; I(POD1_t_) and I(POD2_t_) are indicator variables for POD1 and POD2, respectively. The coefficient β_1_ represents the average adjusted difference between the severe and non-severe groups across POD0–POD2. The coefficients β_2_ and β_3_ represent differences at POD1 and POD2, respectively, relative to POD0. The term u_i_ is the patient-specific random intercept, and ε_i,t_ is the residual error.

Time-specific LMMs were added to the interaction terms between severe desaturation and the postoperative time. This approach allowed the adjusted difference between the severe and non-severe difference to be estimated separately at POD0, POD1, and POD2 using linear contrasts.

$$\mathrm{Scor}e_{i,s,t}=\beta_{0}+\beta_{1}D_{i}+\beta_{2}I(\mathrm{POD}1_{t})+\beta_{3}I(\mathrm{POD}2_{t})+\gamma_{1}D_{i}I(\mathrm{POD}1_{t})+\gamma_{2}D_{i}I(\mathrm{POD}2_{t})+\beta_{4}\mathrm{Ag}e_{i}+\beta_{5}\mathrm{Weigh}t_{i}+\beta_{6}\mathrm{Se}x_{i}+\beta_{7}\mathrm{RACH}S_{i}+\beta_{8}\mathrm{CPBtim}e_{i}+u_{i}+\varepsilon_{i,t}$$

In this time-specific model, γ_1_ and γ_2_ are the interaction coefficients for severe desaturation at POD1 and POD2, respectively. The adjusted group difference at POD0 is β_1_, that at POD1 is β_1_ + γ_1_, and that at POD2 is β_1_ + γ_2_. The remaining parameters are defined as described above.

GEEs were used to analyse the robustness of the repeated-measures results. GEE models use a Gaussian family, identity link, exchangeable working correlation, and patient ID as the clustering variables. The population-averaged model was as follows:

$$E(\mathrm{Scor}e_{i,s,t}|X_{i,t})=\beta_{0}+\beta_{1}D_{i}+\beta_{2}I(\mathrm{POD}1_{t})+\beta_{3}I(\mathrm{POD}2_{t})\;+\;\beta_{4}\mathrm{Ag}e_{i}+\beta_{5}\mathrm{Weigh}t_{i}+\beta_{6}\mathrm{Se}x_{i}+\beta_{7}\mathrm{RACH}S_{i}+\beta_{8}\mathrm{CPBtim}e_{i}$$

Here, E(Score_i,s,t_ | X_i,t_) denotes the expected composite score conditional on the covariate vector X_i,t_. The vector X_i,t_ includes the severe desaturation status, postoperative time indicators, age, body weight, sex, RACHS-1 score, and CPB time. In the GEE model, β_1_ represents the population-averaged adjusted difference between the severe and non-severe groups. The other coefficients have the same meanings as in the LMM.

Missing values were handled by performing a complete-case analysis within each model. False discovery rate (FDR) control was performed using the Benjamini‒Hochberg procedure. For overall analyses of the LMMs and GEEs, *P* values were adjusted separately by model type across the inflammation and brain injury systems. For time-specific analyses of the LMMs and GEEs, *P* values were adjusted within each biological system and model type. For multivariable linear regression analyses of composite and individual biomarker outcomes, *P* values were adjusted across the outcomes tested within the corresponding analysis.

### 2.6 Software

Statistical analyses were conducted in R (version 4.5.2) (27) using lme4 (28), lmerTest (29), sandwich (30), geepack (31) and emmeans (32), as appropriate; figures were created with ggplot2 (33).

## 3 Results

### 3.1 Study cohort and perioperative characteristics

The study included 104 children, of whom 80 (76.9%) were categorized as having non-severe ΔrScO_2_-max, and 24 (23.1%) were categorized as having severe ΔrScO_2_-max. Overall, 44 of the 104 children (42.3%) were male, the median age was 8.00 months, the median height was 68 cm, and the median body weight was 7.40 kg (Table 1). The average age of patients in the severe desaturation group was 15.1 ± 32.0 months, and the average age of patients in the non-severe group was 32.7 ± 47.8 months. Children in the severe desaturation group were younger than those in the non-severe group (*P* = 0.027). The correlation analysis between age and ΔrScO_2_-max showed a significant negative correlation between age and ΔrScO_2_-max (Pearson's r = −0.261; *P* = 0.008). The younger the age was, the greater the ΔrScO_2_-max was. Other baseline and perioperative variables, including sex, height, weight, RACHS-1 score, disease status, haemoglobin level before CPB, oxygen partial pressure before CPB, haemoglobin level after CPB, surgery time, CPB time, ACC time, anaesthesia duration, postoperative mechanical ventilation duration, intensive care unit stay, and hospital stay, did not differ significantly between the two groups.

**Table 1:** Clinical characteristics according to ΔrScO_2_-max severity.

Variable	Overall *n* = 104	Non-severe ΔrScO_2_-max *n* = 80	Severe ΔrScO_2_-max *n* = 24	*P* value
**1. Basic information**				
Sex, male	44/104 (42.3%)	37/80 (46.2%)	7/24 (29.2%)	0.163
Age (month)	8.00 [2.00, 27.00]	9.50 [2.00, 48.00]	3.00 [1.00, 12.00]	**0** **.** **027**
Height (cm)	68.00 [54.50, 88.50]	69.00 [55.50, 99.00]	64.00 [52.75, 79.00]	0.158
Weight (kg)	7.40 [4.15, 14.00]	7.60 [4.47, 14.50]	5.50 [3.95, 9.02]	0.127
RACHS-1 score	2.00 [2.00, 2.00]	2.00 [2.00, 2.00]	2.00 [2.00, 2.00]	0.516
Disease, *n* (%)				0.845
Ventricular septal defect	63 (60.6%)	47 (58.8%)	16 (66.7%)	
Atrial septal defect	15 (14.4%)	12 (15.0%)	3 (12.5%)	
Pulmonary valve stenosis	13 (12.5%)	11 (13.8%)	2 (8.3%)	
Coarctation of the aorta	5 (4.8%)	3 (3.8%)	2 (8.3%)	
Mitral valve insufficiency	1 (1.0%)	1 (1.2%)	0 (0.0%)	
Endocardial cushion defect	2 (1.9%)	2 (2.5%)	0 (0.0%)	
Total anomalous pulmonary venous connection	2 (1.9%)	1 (1.2%)	1 (4.2%)	
Tricuspid valve insufficiency	1 (1.0%)	1 (1.2%)	0 (0.0%)	
Tetralogy of Fallot	2 (1.9%)	2 (2.5%)	0 (0.0%)	
**2. Intraoperative variables**				
Hemoglobin before CPB (g/dL)	11.50 [10.10, 12.40]	11.45[10.02, 12.35]	11.75 [10.43, 13.38]	0.217
Oxygen partial pressure before CPB (mmHg)	228.00 [151.50, 267.75]	232.00 [163.50, 292.50]	220.50 [172.00, 286.00]	0.128
Hemoglobin after CPB (g/dL)	12.30 [11.10, 13.20]	12.40 [11.00, 13.27]	12.15 [11.50, 12.68]	0.619
Surgery time (min)	148.50 [130.00, 175.00]	145.00 [130.00, 179.25]	150.00 [133.75, 175.00]	0.526
CPB time (min)	54.00 [43.00, 71.00]	53.00 [41.00, 71.00]	59.00 [49.75, 73.00]	0.323
ACC time (min)	27.00 [18.75, 38.25]	26.50 [18.00, 38.00]	30.50 [23.75, 42.00]	0.285
Anesthesia time (min)	212.50 [185.00, 246.25]	215.00 [185.00, 253.75]	210.00 [183.75, 240.00]	0.997
**3. Clinical outcomes**				
Postoperative mechanical ventilation duration (h)	73.00 [19.75, 135.25]	70.00 [15.75, 137.00]	81.50 [26.50, 117.00]	0.938
ICU stay (day)	5.50 [2.00, 8.00]	5.00 [2.00, 8.00]	6.00 [2.00, 7.50]	0.901
Hospital stay (day)	15.00 [11.00, 20.00]	16.00 [11.00, 20.25]	14.00 [12.00, 18.00]	0.435
Postoperative hospital stay (day)	12.00 [8.75, 16.00]	12.50 [8.00, 17.00]	11.00 [9.00, 14.25]	0.326
POD0 urine output (mL/kg/h)	4.30 [3.89, 5.20]	4.27 [3.89, 5.25]	4.68 [4.09, 5.16]	0.495
Peritoneal dialysis	2/104 (1.9%)	2/80 (2.5%)	0/24 (0.0%)	1.000
Postoperative CRRT	1/104 (1.0%)	1/80 (1.2%)	0/24 (0.0%)	1.000

Data are presented as median [IQR] for continuous variables and *n*/*N* (%) for categorical variables. *P* values compare the Non-severe and Severe ΔrScO_2_-max groups and were calculated using the Wilcoxon rank-sum test for continuous variables and Fisher's exact test for categorical variables. ACC, aortic cross-clamping; CPB, cardiopulmonary bypass; CRRT, continuous renal replacement therapy; ICU, intensive care unit; IQR, interquartile range; POD0, postoperative day 0; RACHS-1, Risk Adjustment for Congenital Heart Surgery-1; rScO_2_, regional cerebral oxygen saturation.

Bold values indicate statistical significance (*P* < 0.05).

### 3.2 Postoperative increases in the levels of inflammatory cytokines and biomarkers of brain injury following CPB

**Figure 1:**
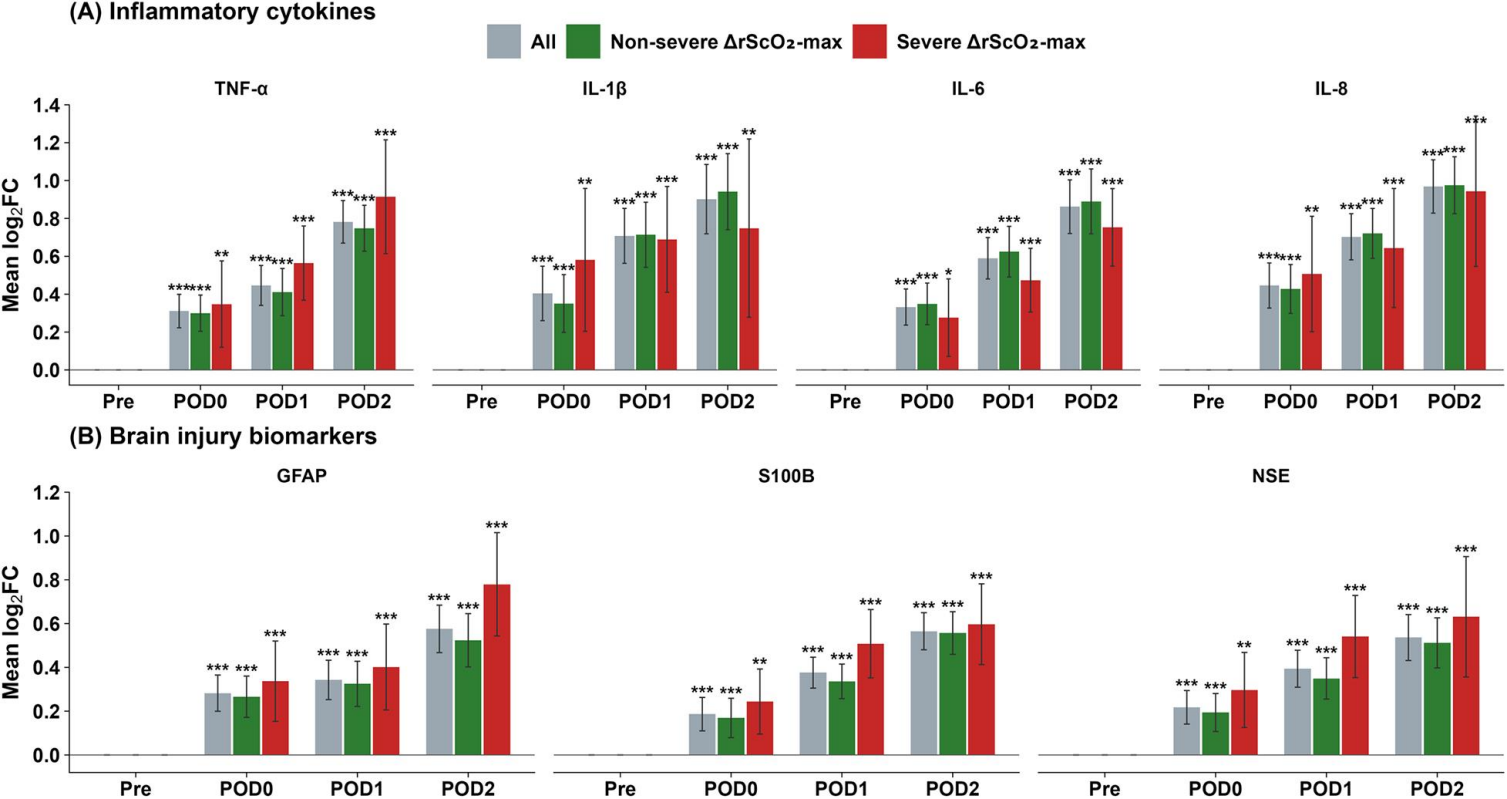
Postoperative elevation in both inflammatory cytokines and brain injury biomarkers. Preoperative values (Pre) were used as the baseline. For each cytokine and brain injury biomarker, log_2_ fold changes at POD0, POD1, and POD2 were calculated relative to Pre using the formula log_2_FC = log_2_(POD/Pre). Bars represent the mean log_2_ fold change at each postoperative time point, and error bars indicate the 95% confidence interval (95% CI) of the mean. Differences between each postoperative time point and Pre were assessed using paired Student's *t*-tests. Statistical significance is indicated as follows: *, *P* < 0.05; **, *P* < 0.01; ***, *P* < 0.001. Sample sizes were *n* = 104 for Pre, POD0, and POD1, and *n* = 93 for POD2.

Figure 1 shows the postoperative changes in the levels of inflammatory cytokines and brain injury biomarkers stratified by the severity of intraoperative cerebral oxygen desaturation. Postoperative changes are reported as the mean log_2_ fold changes relative to the preoperative baseline values, with Pre fixed at 0.

In terms of inflammatory cytokines, TNF-α, IL-1β, IL-6, and IL-8 levels generally increased after surgery, with significant increases in the levels of several cytokines at POD0, POD1, or POD2 compared with the Pre levels. These findings suggest that inflammation occurred early after surgery and persisted during the early postoperative period. The severe ΔrScO_2_-max group showed postoperative inflammatory responses comparable to those observed in the overall cohort and the non-severe group.

In terms of brain injury biomarkers, GFAP, S100B, and NSE levels also increased postoperatively to varying degrees compared with their preoperative values. These significant postoperative elevations suggest that the perioperative period of congenital cardiac surgery is associated with measurable changes in the circulating levels of brain injury biomarkers. However, the presence of significant changes within both the non-severe and severe ΔrScO_2_-max groups indicates that the postoperative increases in the levels of these biomarkers were not restricted to patients with severe intraoperative cerebral oxygen desaturation.

Overall, the results demonstrated that the levels of both inflammatory cytokines and brain injury biomarkers increased after CPB, and these changes were observed in all groups rather than being restricted to the severe ΔrScO_2_-max group. Therefore, based on the ELISA results alone, severe ΔrScO_2_-max appears to identify patients with greater intraoperative cerebral oxygen desaturation, but the results primarily support postoperative within-group changes rather than definitive between-group differences.

### 3.3 Perioperative rScO_2_ trajectories and cerebral desaturation

**Figure 2:**
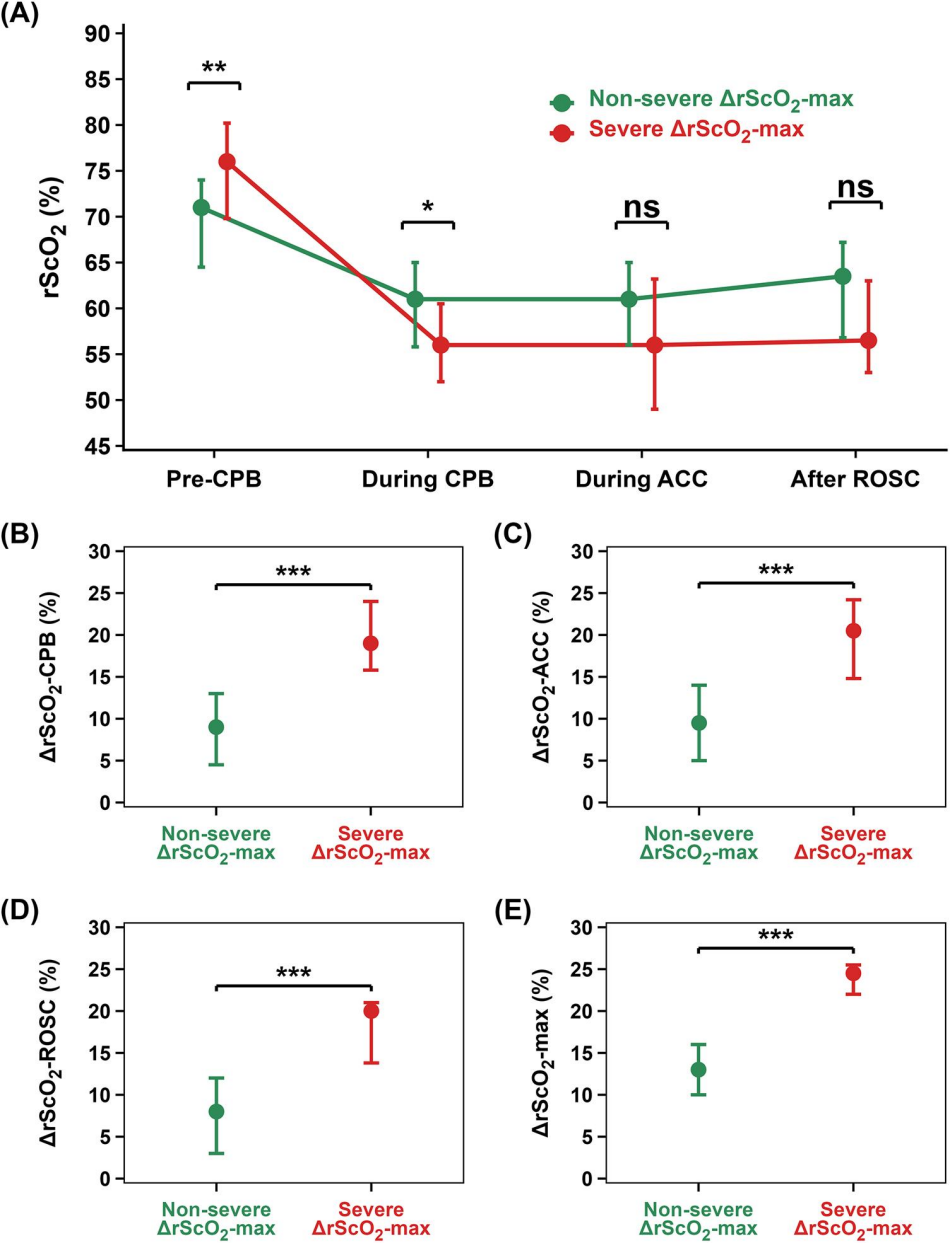
Intraoperative rScO_2_ and ΔrScO_2_ profiles. **(A)** shows the trajectory of rScO_2_ (%) at Pre-CPB, During CPB, During ACC, and After ROSC in the Non-severe ΔrScO_2_-max group and the Severe ΔrScO_2_-max group. Points represent medians, and error bars represent interquartile ranges. **(B**–**E)** show between-group differences in ΔrScO_2_-CPB, ΔrScO_2_-ACC, ΔrScO_2_-ROSC, and ΔrScO_2_-max, respectively. Significance is denoted by stars: *P < 0.05, **P < 0.01, ***P < 0.001; ns indicates no statistical significance.

The perioperative rScO_2_ profiles differed between the groups. The trajectories of rScO_2_ suggested that severe desaturation was characterized by a marked intraoperative decrease in rScO_2_ after CPB initiation rather than by a lower pre-CPB baseline value. The pre-CPB rScO_2_ was higher in the severe desaturation group than in the non-severe group (*P* = 0.001). After the initiation of CPB, the rScO_2_ decreased more prominently in the severe group, whose values decreased during CPB (*P* = 0.030). Both during the ACC and after the ROSC, the between-group differences approached statistical significance (during the ACC: *P* = 0.054; after the ROSC: *P* = 0.055), suggesting a persistent trend towards lower rScO_2_ values in the severe desaturation group throughout the later intraoperative period (Figure 2A). Moreover, the severe group showed significantly greater reductions in all ΔrScO_2_ indices compared with the non-severe group. All between-group differences were statistically significant (all *P* < 0.001) (Figures 2B–E).

### 3.4 Adjusted associations with composite inflammation and brain injury scores

The levels of inflammatory cytokines and brain injury biomarkers were repeatedly measured at POD0, 1, and 2. For each of these markers, postoperative changes are reported as log_2_ fold changes relative to the preoperative baseline value. LMMs and GEE models were used as complementary approaches to evaluate the association between ΔrScO_2_-max and longitudinal postoperative changes in the levels of inflammatory cytokines and brain injury biomarkers. LMMs were used to account for within-patient correlations and to estimate subject-specific longitudinal associations, whereas GEE models were applied to estimate population-averaged associations.

**Figure 3:**
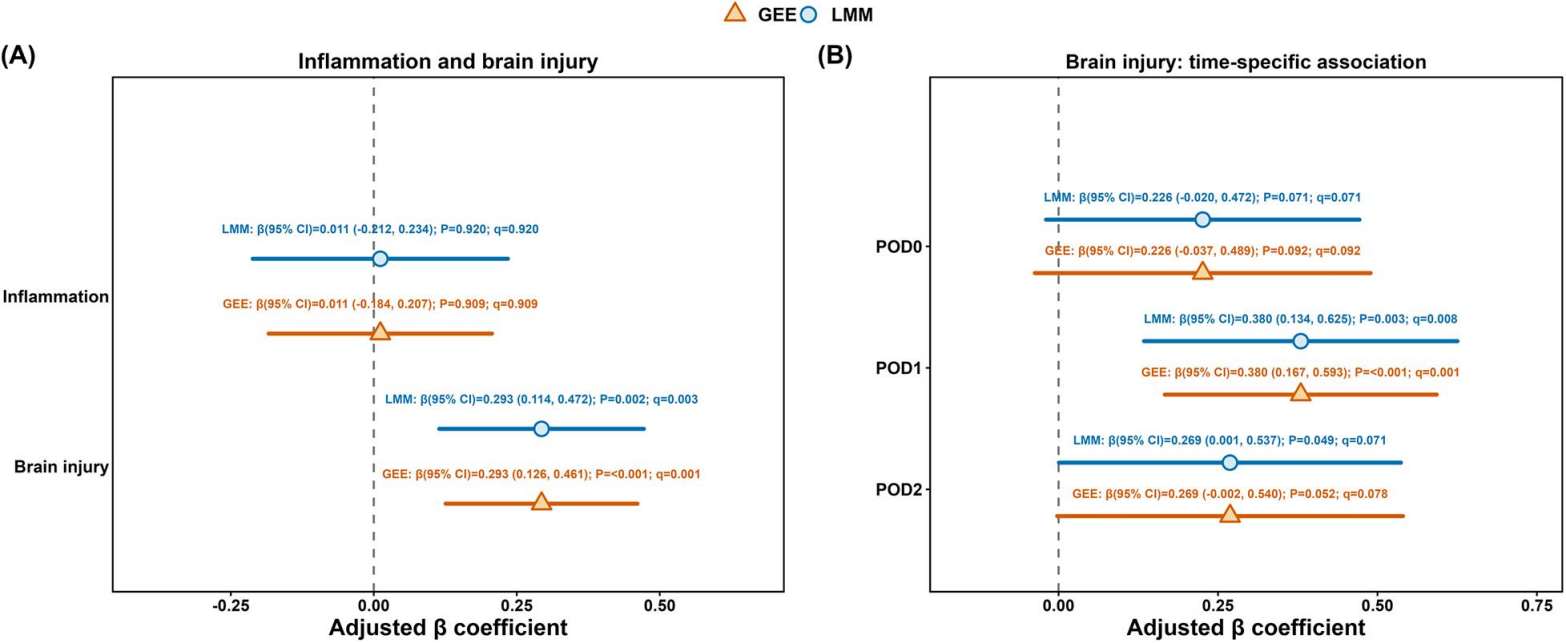
Adjusted associations of severe ΔrScO_2_-max with postoperative inflammatory and brain injury responses estimated using LMM and GEE models. **(A)** Adjusted associations between severe ΔrScO_2_-max and postoperative composite inflammation and brain injury scores. **(B)** Time-specific adjusted associations between severe ΔrScO_2_-max and the composite brain injury score at POD0, POD1, and POD2. Points indicate adjusted β coefficients, and horizontal lines indicate 95% confidence intervals. The vertical dashed line represents no association. Positive β values indicate higher brain injury composite scores in the severe ΔrScO_2_-max group. Models were adjusted for age, body weight, sex, RACHS-1 score, cardiopulmonary bypass time, and postoperative time point where appropriate. q values were calculated using Benjamini–Hochberg FDR correction.

According to the results of the repeated-measures analyses, severe ΔrScO_2_-max was not associated with the postoperative composite inflammation score. The adjusted differences between the severe and non-severe groups were 0.011 in both the LMM (95% CI, −0.212 to 0.234; *P* = 0.920; q = 0.920) and the GEE model (95% CI, −0.184 to 0.207; *P* = 0.909; q = 0.909). In contrast, severe ΔrScO_2_-max was independently associated with a higher postoperative composite brain injury score. The adjusted differences between the severe and non-severe groups were 0.293 in the LMM (95% CI, 0.114–0.472; *P* = 0.002; q = 0.003) and 0.293 in the GEE model (95% CI, 0.126–0.461; *P* < 0.001; q = 0.001) (Figure 3A).

Furthermore, time-specific analyses of LMMs and GEEs consistently indicated positive associations between severe ΔrScO_2_-max and higher brain injury composite scores, with the most robust association occurring at POD1. These findings suggest that severe intraoperative cerebral oxygen desaturation may be associated with increased postoperative release of brain injury biomarkers, particularly on POD1. The adjusted differences between the severe and non-severe groups in the composite brain injury score at POD1 were 0.380 in the LMM (95% CI, 0.134–0.625; *P* = 0.003; q = 0.008) and 0.380 in the GEE model (95% CI, 0.167–0.593; *P* < 0.001; q = 0.001) (Figure 3B). At POD2, the LMM estimate was nominally significant but did not remain significant after FDR correction (*P* = 0.049, q = 0.071). The corresponding GEE estimate at POD2 was directionally similar but not statistically significant after correction (*P* = 0.052; q = 0.078).

Taken together, the results of the longitudinal adjusted analyses revealed a selective association between severe ΔrScO_2_-max and postoperative composite brain injury responses, with no corresponding association for the composite inflammation score.

### 3.5 Composite score distribution and analyses of single biomarkers of brain injury

**Figure 4:**
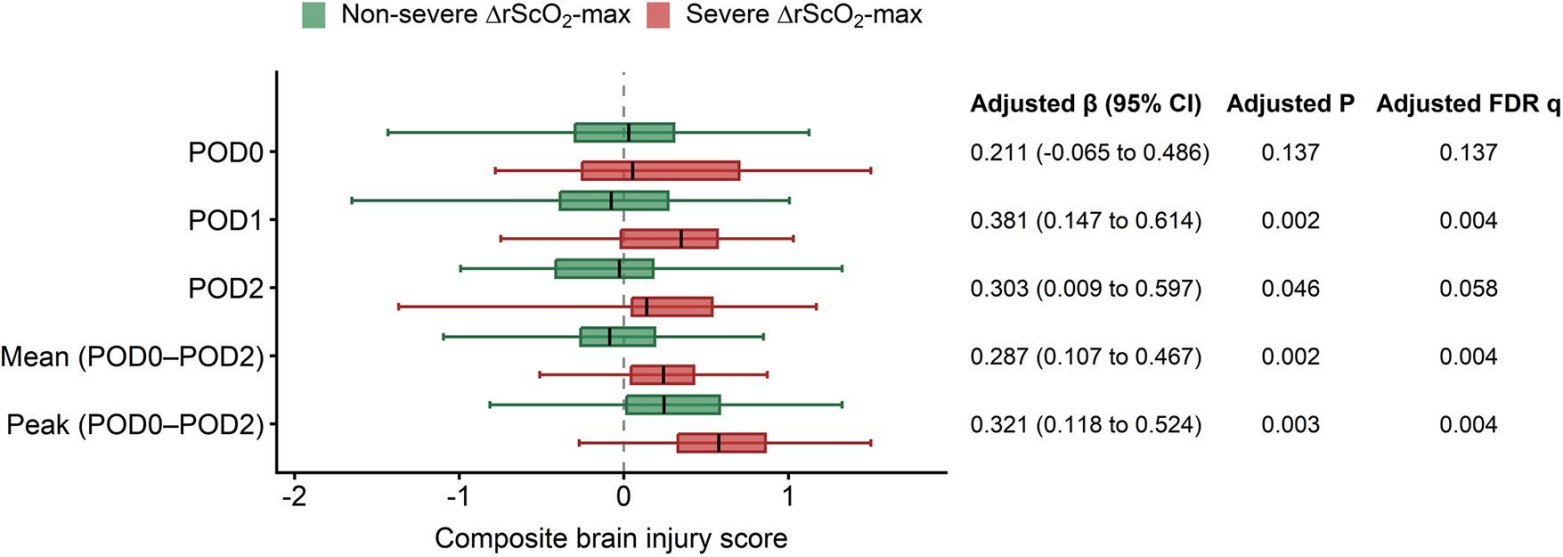
Box-forest plot of composite brain injury scores according to severe cerebral desaturation status. Composite brain injury scores at POD0, POD1, and POD2, as well as the mean and peak composite brain injury scores across POD0–POD2, are shown for patients with non-severe and severe cerebral desaturation. Patients were stratified according to severe cerebral desaturation status, defined by the maximum intraoperative decrease in regional cerebral oxygen saturation (ΔrScO_2_-max). Green boxes represent the non-severe ΔrScO_2_-max group, and red boxes represent the severe ΔrScO_2_-max group. Boxes indicate the interquartile range (IQR), the vertical line within each box indicates the median, and whiskers indicate the minimum and maximum values. The dashed vertical line indicates the reference value of 0 for the composite brain injury score.

The results of distributional and cross-sectional analyses were consistent with the results of the repeated-measures analysis. Children with severe ΔrScO_2_-max had higher median composite brain injury scores at POD1 and POD2 and for the mean and peak POD0–POD2 summary measures (Figure 4). In adjusted linear models with HC3 robust standard errors, severe ΔrScO_2_-max was associated with higher brain injury scores at POD1 (β = 0.381; 95% CI, 0.147–0.614; *P* = 0.002; q = 0.004), higher mean brain injury scores (β = 0.287; 95% CI, 0.107–0.467; *P* = 0.002; q = 0.004), and higher peak brain injury scores (β = 0.321; 95% CI, 0.118–0.524; *P* = 0.003; q = 0.004).

**Figure 5:**
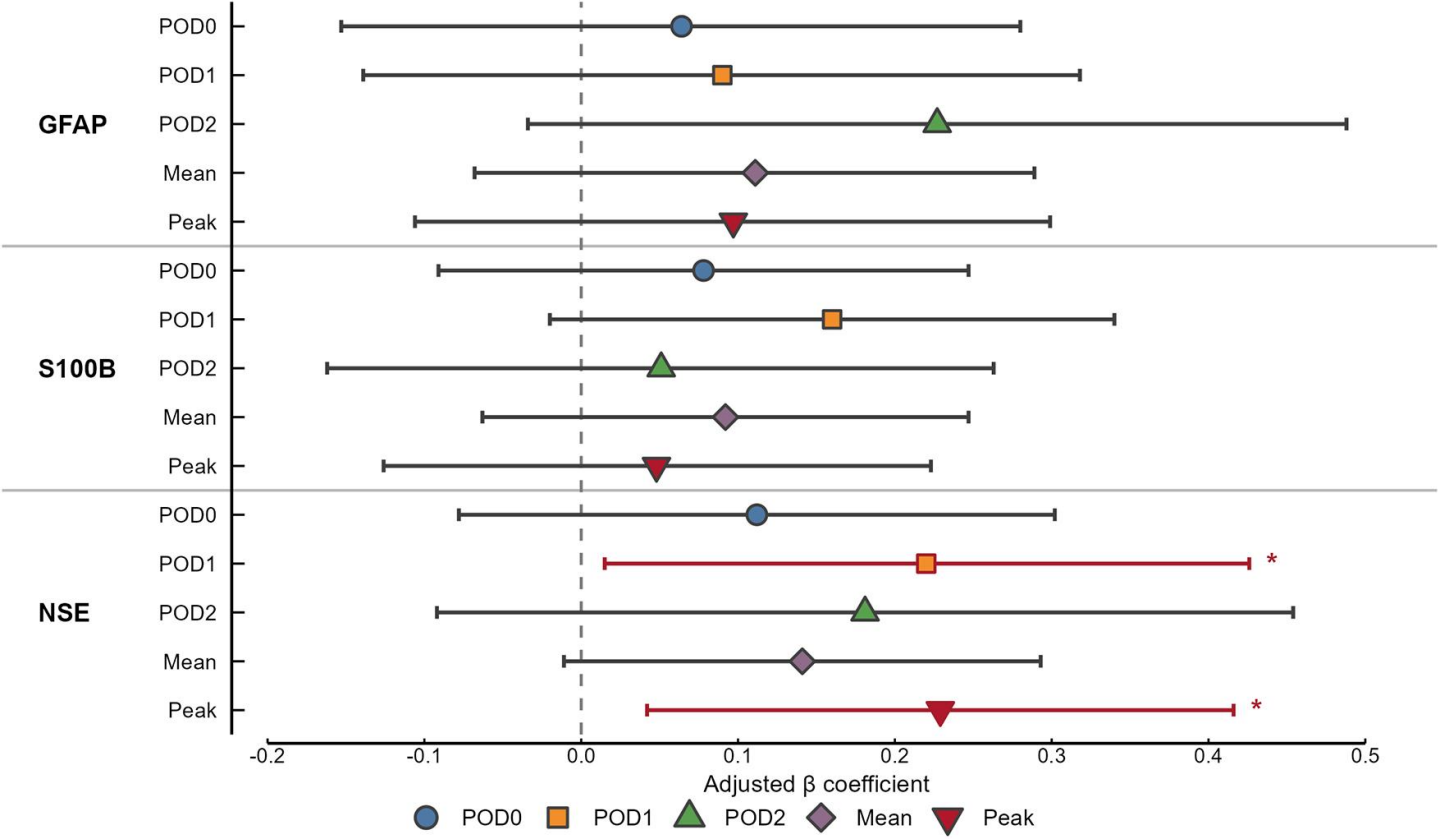
Adjusted associations between severe cerebral oxygen desaturation and perioperative brain injury biomarkers. The forest plot displays adjusted β coefficients and 95% confidence intervals (CIs) for the associations of severe cerebral oxygen desaturation, defined as ΔrScO_2_-max ≥20%, with log_2_ fold changes in GFAP, S100B, and NSE. Estimates are shown for POD0, POD1, POD2, mean values, and peak values. Distinct symbols and colors indicate the five time-derived biomarker metrics. Asterisks indicates estimates with nominal statistical significance based on adjusted *P* < 0.05. Models were adjusted for age, body weight, sex, RACHS-1 score, and CPB time.

Analyses of single biomarkers of brain injury revealed directionally positive estimates for most GFAP, S100B, and NSE outcomes in the severe ΔrScO_2_-max group (Figure 5). NSE showed nominally significant adjusted associations at POD1 (β = 0.220; 95% CI, 0.015–0.426; *P* = 0.038) and for the peak value (β = 0.229; 95% CI, 0.042–0.416; *P* = 0.018), although these single-brain injury biomarker associations did not remain significant after FDR correction.

Overall, the cross-sectional and biomarker analyses supported the longitudinal findings, indicating a stronger association of severe ΔrScO_2_-max with composite brain injury responses than with any single biomarker after correction for multiple analyses.

## 4 Discussion

Children with CHD are being increasingly recognized as a population at risk for adverse neurodevelopmental outcomes, even as surgical survival has improved substantially (4). Contemporary scientific statements emphasize that the neurodevelopmental risk in CHD patients is multifactorial and may be influenced by genetic, foetal, surgical, perioperative, and postoperative factors.

In this single-centre cohort, we found that a maximum intraoperative decrease in rScO_2_ of at least 20% was associated with a greater postoperative composite brain injury biomarker response in children who underwent congenital heart surgery with CPB. This association was observed in repeated-measures LMMs and remained consistent in GEE analyses, supporting the robustness of the findings across different approaches for handling correlated repeated postoperative measurements. The strongest adjusted difference was observed at POD1, suggesting that the biomarker signal may peak after the immediate postoperative period.

The association between severe cerebral desaturation and higher brain injury biomarker scores is biologically plausible. rScO_2_ reflects the balance between cerebral oxygen delivery and oxygen utilization. A marked intraoperative decrease from the pre-CPB value may indicate a period of impaired cerebral oxygen supply, increased extraction, or altered perfusion during the most physiologically stressful stages of cardiac surgery. These conditions could increase the vulnerability of the developing brain, particularly in infants and children undergoing CPB.

Notably, severe ΔrScO_2_-max was not associated with the composite inflammation score. Systemic inflammation after CPB is common and may be driven by blood contact with the CPB circuit, ischaemia‒reperfusion injury, surgical trauma, and endothelial activation (34). Postoperative increases in cytokine levels may represent a generalized CPB-related response rather than a signal specific to rScO_2_. The lack of an association with the composite inflammatory response suggests that the link between severe cerebral desaturation and biomarkers of brain injury may not simply reflect a stronger global inflammatory response. Instead, ΔrScO_2_-max may capture vulnerability more specifically related to cerebral oxygenation instability.

The timing of the association also deserves attention. The greatest difference in brain injury scores appeared on POD1 rather than immediately at POD0. This pattern may reflect the release kinetics, distribution, and clearance of circulating brain injury biomarkers. Samples collected at very early time points may precede maximal peripheral accumulation, whereas samples collected at later time points may better capture downstream cellular injury processes. The estimates obtained on POD2 remained directionally positive, although the statistical strength was attenuated after adjustment for multiple testing.

In research investigating paediatric patients with CHD who are undergoing surgery, neural markers such as GFAP, S100B, and NSE are used to evaluate acute brain injury and the short-term neurodevelopmental risk (15, 18, 35). A composite brain injury score may therefore be advantageous in this setting. Individual biomarkers, such as GFAP, S100B, and NSE, partly reflect different cellular sources and injury-related processes. However, these biomarkers exhibit significant heterogeneity (18, 35). A composite score may therefore capture a broader postoperative brain injury biomarker response and reduce reliance on any single marker. This result may explain why the composite brain injury score was more consistently associated with severe ΔrScO_2_-max than were the levels of individual biomarkers after correction for multiple testing.

The results also emphasize the importance of considering changes from baseline values, not only absolute rScO_2_ values. In this cohort, the severe desaturation group had a higher pre-CPB rScO_2_ but a lower intraoperative nadir, resulting in a higher ΔrScO_2_-max. These findings suggest that children with apparently acceptable baseline saturations may still experience clinically meaningful intraoperative cerebral oxygenation instability. The measurement of the maximum decrease may therefore provide complementary information to absolute saturation thresholds.

Children in the severe desaturation group were younger than those in the non-severe group. The correlation analysis between age and ΔrScO_2_-max revealed a significant negative correlation between age and ΔrScO_2_-max (Pearson's r = −0.261; *P* = 0.008). The younger the age was, the greater the ΔrScO_2_-max was. This age difference may further inform the interpretation of the severe desaturation phenotype. The younger age of patients in this group may be clinically relevant. Neonates and infants have higher cerebral metabolic demands and less mature cerebrovascular regulation, which may increase their susceptibility to hypoxic–ischaemic stress during CPB (5, 11). However, a younger age should not be interpreted simply as a marker of lower baseline cerebral oxygenation because rScO_2_ in paediatric patients is influenced by multiple factors, including the device algorithm, haemoglobin concentration, arterial oxygen saturation, cardiac anatomy, and perioperative conditions.

This study has several limitations. First, it was conducted at a single centre, and the sample size was small, especially for the severe desaturation group. Second, this study only examined the maximum decrease in rScO_2_ but ignored the desaturation burden and duration of desaturation. In future research, we will pay close attention to indicators such as the duration of desaturation and the cumulative desaturation burden to address the shortcomings of our current study. Third, this study only evaluated the levels of biomarkers, which are indirect surrogate measurements and cannot independently determine the presence, severity or clinical significance of nerve damage. This study did not include any direct indicators of neurological function, neuroimaging or neurodevelopmental outcomes, which limited the clinical application value of the research results. Future studies should integrate continuous NIRS-derived metrics with perioperative haemodynamic data, neuroimaging, electroencephalography, and longitudinal neurodevelopmental follow-up. Fourth, residual confounding factors are an important issue. We have made every effort to control for confounding and have conducted multivariate adjustments. However, as this study was a preliminary exploratory study with a small sample size, many confounding factors could not be excluded. Next, we should conduct a study with a larger sample size, involving multiple centres, and with more detailed control over confounding factors to ensure that the research results are more accurate and clinically significant. Fifth, this study was dominated by relatively low-risk procedures, with a median RACHS-1 score of 2, and the results might not apply to patients with more complex CHDs, which affected the applicability of the results of this study.

## 5 Conclusions

In children who underwent congenital heart surgery with CPB, severe intraoperative cerebral desaturation, defined as a ΔrScO_2_-max of at least 20%, was more common in younger children and was independently associated with a greater postoperative composite brain injury biomarker response. This association showed a time-dependent pattern: the difference between the severe and non-severe desaturation groups was most evident on POD1, was less pronounced immediately after surgery on POD0, and remained directionally positive but attenuated by POD2.

## Data Availability

The datasets generated and/or analyzed during the current study are available from the corresponding author on reasonable request. The data are not publicly available due to privacy and ethical restrictions involving pediatric patient information.
